# Vogt–Koyanagi–Harada disease following BCG vaccination and tuberculosis

**DOI:** 10.1186/s40064-016-2223-4

**Published:** 2016-05-12

**Authors:** Berna Dogan, Muhammet Kazim Erol, Ayse Cengiz

**Affiliations:** Department of Ophthalmology, Antalya Training and Research Hospital, Varlık Mh., Kazim Karabekir Caddesi, 07100 Antalya, Turkey

**Keywords:** BCG vaccine, Serous retinal detachment, Tuberculosis, Vogt–Koyanagi–Harada disease

## Abstract

**Introduction:**

To describe the characteristics, diagnosis, and treatment of the first documented case of Vogt–Koyanagi–Harada (VKH) disease following BCG vaccination (Patient 1) and the first documented case of both VKH disease and tuberculosis (Patient 2).

Two patients were diagnosed with VKH disease and monitored using fundus photography, fundus autofluorescence, fluorescein angiography (FA), spectral-domain optical coherence tomography, and enhanced depth imaging optical coherence tomography (EDI-OCT).

**Case description:**

A 39-year-old patient (Patient 1) had bilateral granulomatous anterior uveitis and serous retinal detachment. FA showed multiple punctuate hyperfluorescent lesions and multilobular pools of dye. EDI-OCT revealed serous retinal detachment, subretinal septa, and cystoid spaces. A 40-year-old woman (Patient 2) presented with a 3-week history of decreased vision, headache and tinnitus. Fundus examination showed bilateral disc swelling with serous retinal detachment and retinal folds. She had been diagnosed with tuberculosis. EDI-OCT showed fluctuation of the internal limiting membrane (ILM), retinal folds, retinal pigment epithelial (RPE)-Bruch membrane undulation, choroidal folds, serous retinal detachment. Both of the patients received high dosage of steroid treatment during the diagnosis. A fast recovery in VKH symptoms was observed following the treatment.

**Discussion and evaluation:**

Immunological mechanisms and dysregulation of the immune system may play a significant role in the association between VKH disease and BCG.

**Conclusions:**

EDI-OCT imaging demonstrated structural changes in the photoreceptor layer, RPE-Bruch membrane, choroid, outer retina, ILM in acute VKH.

## Background

Vogt–Koyanagi–Harada (VKH) disease is a rare granulomatous inflammatory disorder affecting the eyes, auditory system, meninges and skin. The precise etiology and pathogenesis of VKH disease are unknown, but current clinical and experimental evidence suggests a cell-mediated autoimmune process driven by T-lymphocytes directed against self-antigens associated with melanocytes in genetically susceptible individuals (Moorthy et al. 1995; Rao 1997). Typical clinical features of VKH include bilateral panuveitis associated with exudative retinal detachment, meningism associated with headache and pleocytosis of cerebrospinal fluid, tinnitus or hearing loss, and cutaneous changes, such as alopecia, poliosis, and vitiligo. Presence of ocular and two or more extraocular features is considered as a complete form of VKH disease. Incomplete VKH disease includes bilateral typical ocular involvement plus either neurologic/auditory or cutaneous changes, whereas probable VKH disease is composed of just ocular manifestations. However, some of these probable VKH patients can develop cutaneous manifestations during the chronic or chronic recurrent stage of the disease. The acute phase of the VKH disease is characterized by bilateral uveitis, diffuse choroidal inflammation, and multifocal serous retinal detachment (Read et al. 2001). During this phase, the neural retina is detached from the retinal pigment epithelium (RPE), the subretinal space is filled with an eosinophilic exudate of proteinaceous material, and the choroid is diffusely infiltrated by lymphocytes, with focal aggregates of epithelioid histiocytes and multinucleated giant cells. These histologic changes during the acute phase have been observed to incur early damage to choroidal melanocytes. Immunohistochemical analysis has also revealed that the choroidal infiltrates observed during this phase are predominantly comprised of T-lymphocytes (Rao 2007).

The chronic phase in which progressive choroidal depigmentation occurs results in a change classically described as sunset glow fundus. During this stage, vitiligo and poliosis may occur (Moorthy et al. 1995; Read et al. 2001), and some patients may develop vision-threatening retinal complications, including choroidal neovascularization and subretinal fibrosis (Read et al. 2001).

Enhanced depth imaging optical coherence tomography (EDI-OCT) and fluorescein angiography (FA) are of great importance in diagnosis of VKH disease, monitoring of morphological changes in the retina and choroid during its course, and evaluation of the efficacy of ophthalmologic therapies (Ishihara et al. 2009). Using EDI-OCT, disruption of the outer retina, especially of the inner/outer segment (IS/OS) junction, and of the cone outer segment tips (COST) line, as well as irregularities of the RPE layer, have been observed in acute VKH disease (Gupta et al. 2009; Vasconcelos-Santos et al. 2010).

## Case description

**Fig. 1:**
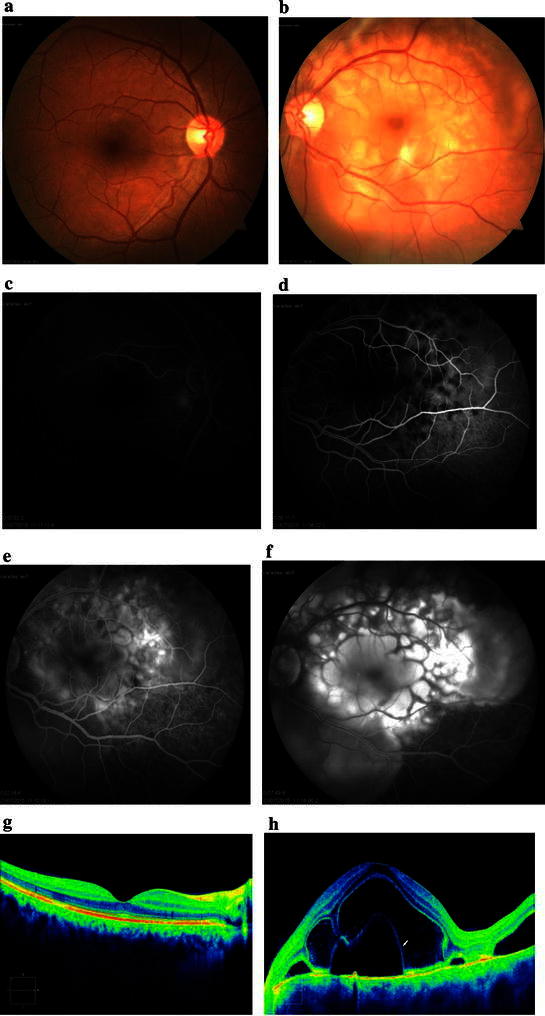
Images of the right eye and left eye of Patient 1 at diagnosis of VKH disease. **a**, **b** Color fundus photograph. *Left eye* shows serous retinal detachment. **c** Early-phase fluorescein angiogram (FA) of the *right eye* shows hyperfluorescent dots. **d** Early-phase FA of the left eye shows multiple punctuate hyperfluorescent lesions on the retinal pigment epithelium (RPE) and multiple small round hypofluorescent lesions, suggesting uneven filling of the choriocapillaris (*dark areas*). **e**, **f** Mid-phase FA (2 min) and late-phase FA (7 min) of the *left eye* shows multilobular pools of dye. Foveal round area of the dye pooling has a dark rim. **g** Enhanced depth imaging optical coherence tomography (EDI-0CT) of the *right eye* shows choroidal hyperreflectivity. Chroidal thickness = 436 µm (OD). **h** EDI-OCT of the *left eye* shows serous retinal detachment, subretinal cystoid spaces, and subretinal septa (*white arrow*) separating the cystoid space from subretinal fluid. The subretinal cystoid spaces are slightly more reflective than the area of serous retinal detachment. Retinal thickness = 1246 µm (OS). Enlargement of the choroid of the *left eye* is not visible on EDI-OCT at this stage

Patient 1 was a 39-year old Caucasian male who had been diagnosed with superficial transitional cell carcinoma (TCC) of the bladder in 2015. He was initially treated with intravesical immunotherapy consisting of weekly instillations of BCG for a total of 4 weeks. After the fourth dosage of BCG treatment had been administered, he was admitted to our clinic complaining of blurred vision in both eyes, headache and malaise. Neurologic examination was nonrevealing. Brain magnetic resonance imaging (MRI) was normal. His angiotensin-converting enzyme (ACE) level was normal, and serological testing for syphilis, Lyme disease, cytomegalovirus (CMV), and toxoplasma was negative. His best-corrected visual acuity (BCVA) was 20/25 in the right eye (OD) and 20/50 in the left eye (OS). Slit-lamp biomicroscopy showed anterior chamber cells. Left eye had serous retinal detachment in the posterior fundus (Fig. 1b). Early-phase FA of the left eye showed multiple punctuate hyperfluorescent lesions on the RPE and multiple small round hypofluorescent lesions, suggesting an uneven filling of the choriocapillaris (Fig. 1d). Mid-phase FA and late-phase FA of the left eye showed multilobular pools of dye (Fig. 1e, f). EDI-OCT of the left eye revealed serous retinal detachment, subretinal septa, cystoid spaces, and choroidal hyperreflectivity (Fig. 1h). The subretinal septa was a portion of the outer segment (OS) layer separated from the inner segment (IS) layer by cystoid spaces. The subretinal cystoid spaces were mildly reflective, whereas the areas of serous retinal detachment were not reflective.

Based on these observations, the patient was treated with 1 mg/kg/day of oral prednisone that was gradually tapered off over 6 months.

**Fig. 2:**
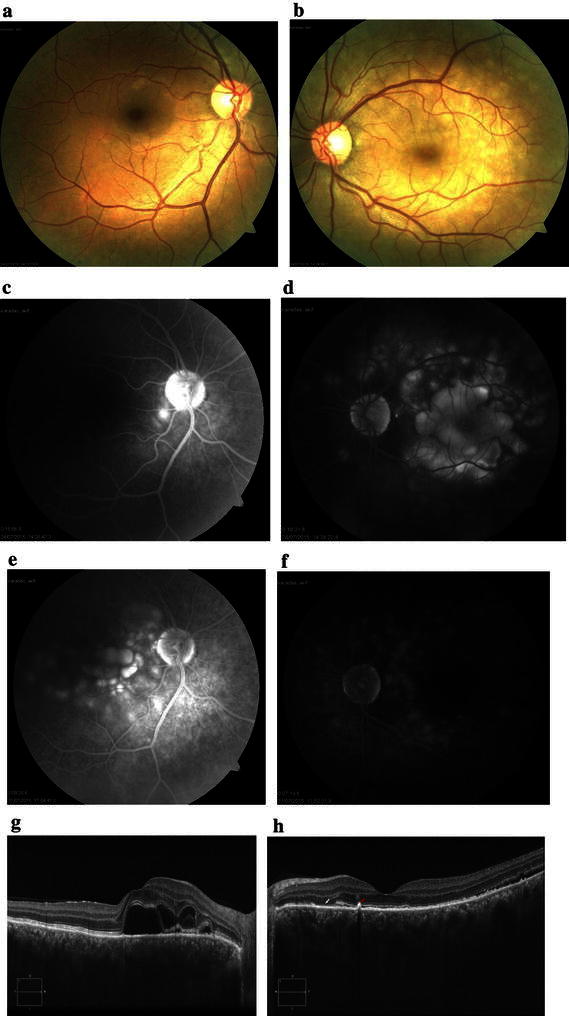
Images of the right eye and left eye of Patient 1. **a**, **b** Four days after initiation of steroid therapy, serous retinal detachment has been resolved in the left eye. **c** Late-phase FA (18 min) of the *right eye* shows hyperfluorescent dots. **d** Late-phase FA (18 min) of the *left eye* shows decrease in multiple punctuate hyperfluorescent lesions and multilobular pools of dye. **e**, **f** Ten days after initiation of steroid therapy, late-phase FA (9 min) of the *right eye* shows increase in multilobular pools of dye. Late-phase FA (7 min) of the *left eye* shows decrease in multiple punctuate hyperfluorescent lesions. **g** EDI-OCT image of the *right eye* shows serous retinal detachment, subretinal cystoid spaces, and subretinal septa separating the cystoid space from subretinal fluid. Chroidal thickness = 564 µm. **h** EDI-OCT image of the *left eye* shows decrease in serous retinal detachment and complete resolution of subretinal cystoid spaces. EDI-OCT reveals hyper-reflective lesions at the level of the retinal pigment epithelium (RPE) with disruption of the inner segment/outer segment (IS/OS) line (Dalen-Fuchs nodule) (*red arrow*) and discontinuous IS/OS line (*white arrow*). Choroidal thickness = 541 µm

**Fig. 3:**
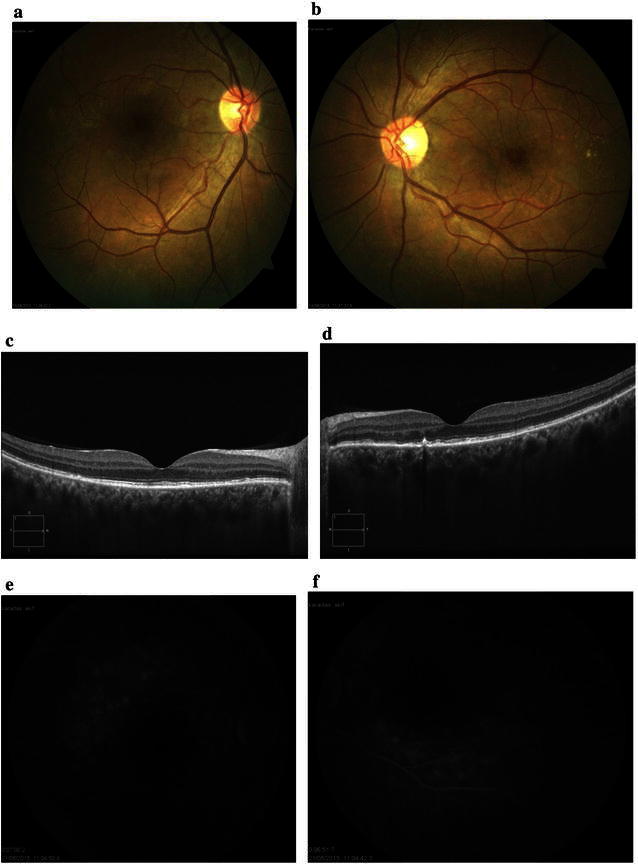
Images of the right eye and left eye of Patient 1. **a**, **b** Color fundus photos 1 month after initiation of steroid therapy. **c**, **d** EDI-OCT 1 month after initiation of steroid therapy. Choroidal thickness = 414 µm (OD) and 458 µm (OS). **e**, **f** FA 1 month after initiation of steroid therapy

Four days after initiation of steroid therapy, the serous retinal detachment was resolved in the left eye (Fig. 2b). Whereas late-phase FA of the right eye showed hyperfluorescent dots (Fig. 2c), late-phase FA of the left eye showed a decrease in multiple punctuate hyperfluorescent lesions and multilobular pools of dye (Fig. 2d). Ten days after initiation of steroid therapy, late-phase FA of the right eye showed an increase in multilobular pools of dye, while late-phase FA of the left eye showed a decrease in multiple punctuate hyperfluorescent lesions and multilobular pools of dye (Fig. 2e, f). EDI-OCT of the right eye showed serous retinal detachment, subretinal cystoid spaces, and subretinal septa separating the cystoid space from subretinal fluid (Fig. 2g). EDI-OCT of the left eye showed a decrease in serous retinal detachment, and the subretinal cystoid spaces completely resolved. EDI-OCT revealed hyper-reflective lesions at the level of the retinal pigment epithelium with disruption of the IS/OS line (Dalen-Fuchs nodule), and disappearance of the COST line and the IS/OS junction and the vertical lining on the outer plexiform layer (Fig. 2h). One month after the start of steroid therapy, best-corrected visual acuity (BCVA) was 20/20 in both eyes (Fig. 3).

Patient 2 was a 40-year old darkly pigmently Caucasian female admitted to our clinic in July 2015 complaining of blurred vision in both eyes and optic disc edema. She had been referred by the neurology clinic, which had been monitoring her for the past 3 weeks for complaints of malaise, headache, and tinnitus. Lumbar puncture revealed sterile cerebrospinal fluid lymphocytic pleocytosis. Level of angiotensin-converting enzyme (ACE) was normal; the results of serological testing for syphilis, Lyme disease, herpes simplex virus, varicella zoster virus, cytomegalovirus, and toxoplasma was negative; and brain magnetic resonance imaging was normal. In her previous medical history, she had been diagnosed with tuberculosis and had been treated for 9 months. At diagnosis, her BCVA was 20/50 in the right eye (OD) and 20/32 in the left eye (OS). Slit-lamp biomicroscopy showed bilateral anterior chamber cells. Intraocular pressure was 13 mm Hg OU.

**Fig. 4:**
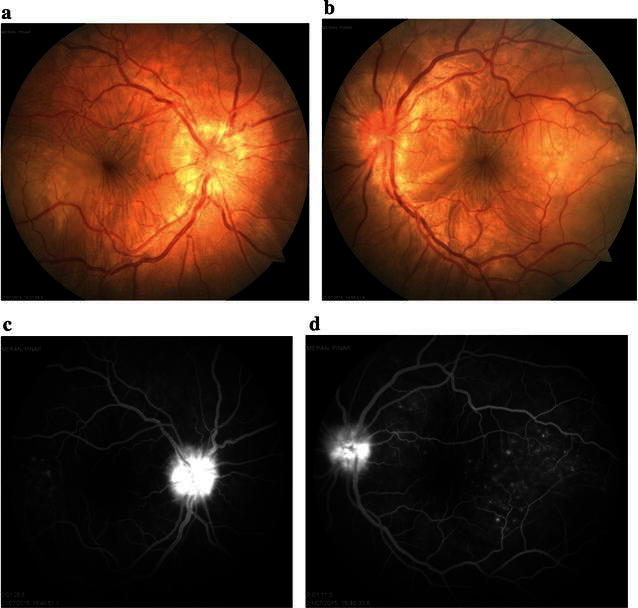
Images of the right and left eyes of Patient 2 at diagnosis of VKH disease. **a**, **b** Color fundus photos show bilateral disc swelling with serous retinal detachment and retinal folds. **c**, **d**: Early-phase FA (1 min) shows multiple punctuate hyperfluorescent lesions on the RPE and optic disc hyperfluorescence

**Fig. 5:**
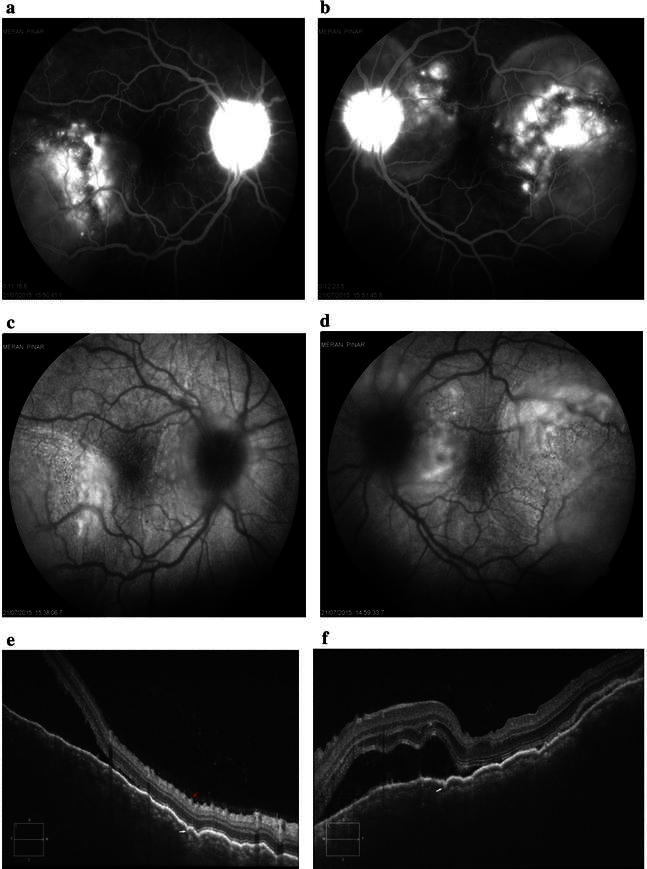
Images of the right and left eyes of Patient 2 at diagnosis of VKH disease. **a**, **b** Late-phase FA (12 min) shows multilobular pools of dye. **c**, **d** Fundus autofluorescence (FAF) reveals mixed hypo-hyperautofluorescence. **e**, **f** EDI-OCT shows fluctuation of internal limiting membrane (ILM) (*red arrow*), retinal folds, RPE-Bruch membrane undulation (*white arrow*), choroidal folds, serous retinal detachment. Retinal thickness = 688 µm (OD) and 641 µm (OS). Enlargement of the choroid of the both eyes is not visible on EDI-OCT at this stage

Fundus examination showed bilateral optic disc swelling with serous retinal detachment and retinal folds (Fig. 4a, b). Early-phase FA showed multiple punctuate hyperfluorescent lesions on the RPE and optic disc hyperfluorescence (Fig. 4c, d). Late-phase FA showed multilobular pools of dye (Fig. 5a, b). Fundus autofluorescence (FAF) revealed mixed hypo-hyperautofluorescence (Fig. 5c, d). EDI-OCT showed fluctuation of internal limiting membrane (ILM), retinal folds, RPE-Bruch membrane undulation, choroidal folds, and serous retinal detachment. The choroid was enlarged and not visible on EDI-OCT at this stage (Fig. 5e, f). Based on these findings, administration of 1 mg/kg/day of prednisone was initiated, and then gradually tapered over 6 months. The effects of the treatment were monitored with weekly EDI-OCT imaging until resolution of serous retinal detachment.

**Fig. 6:**
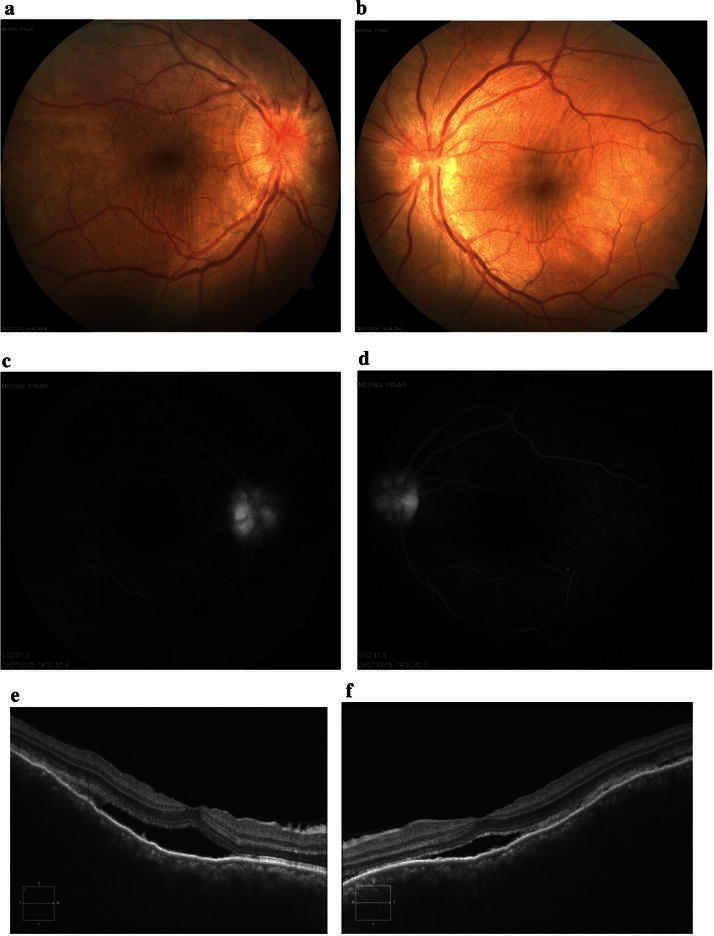
Images of the right and left eyes of Patient 2. **a**, **b** Eight days after initiation of steroid therapy, serous retinal detachment and disc swelling have resolved. **c**, **d** Mid-phase FA (3 min) shows decrease in multiple punctuate hyperfluorescent lesions and multilobular pools of dye. **e**, **f** EDI-OCT shows decrease in fluctuation of ILM, retinal folds, RPE-Bruch membrane undulation, choroidal folds, and serous retinal detachment

**Fig. 7:**
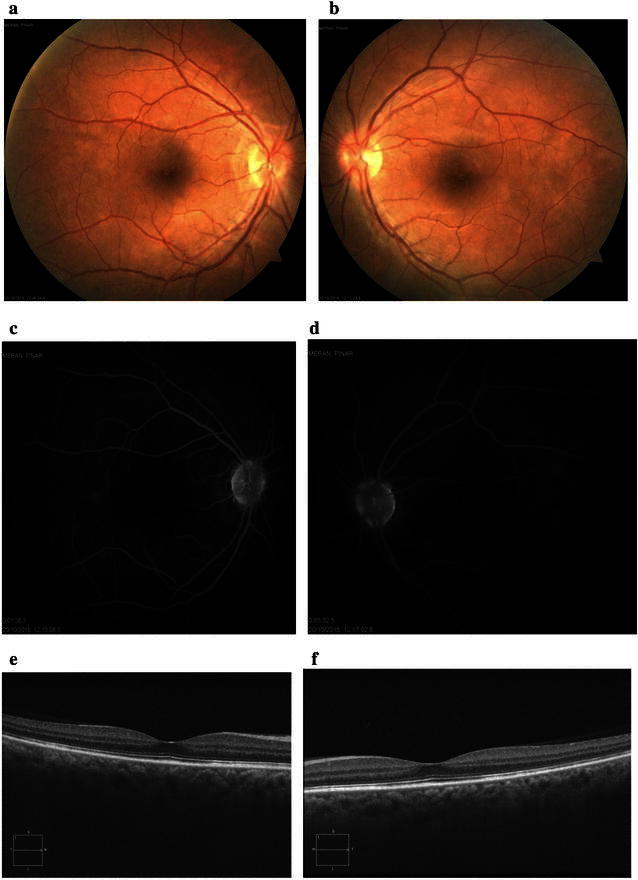
Images of the right and left eyes of Patient 2. **a**, **b** Color fundus photos taken 3 months after initiation of steroid therapy show disappearance of serous retinal detachment with disc swelling and retinal folds. **c**, **d** FA shows minimal optic disc hyperfluorescence. **e**, **f** EDI-OCT shows complete resolution of serous retinal detachment and RPE-Bruch membrane undulation. Choroidal thickness = 365 µm (OD) and 346 µm (OS)

Eight days after initiation of steroid therapy, serous retinal detachment and disc swelling resolved (Fig. 6a, b). Mid-phase FA showed decrease in multiple punctuate hyperfluorescent lesions and multilobular pools of dye (Fig. 6c, d). EDI-OCT showed decrease in fluctuation internal limiting membrane, retinal folds, RPE-Bruch membrane undulation, choroidal folds, and serous retinal detachment (Fig. 6e, f). Three months after the start of steroid therapy, color fundus photos showed disappearance of serous retinal detachment with disc swelling and retinal folds (Fig. 7a, b), FA showed minimal optic disc hyperfluorescence (Fig. 7c, d), and EDI-OCT confirmed complete resolution of serous retinal detachment and RPE-Bruch membrane undulation (Fig. 7e, f). Her best-corrected visual acuity (BCVA) was 20/20 in both eyes. About 2–3 months after the onset of VKH, vitiligo occurred on the face.

## Discussion

The OCT findings of previous research have confirmed that the densest tissue affected by the inflammatory process in VKH disease is the choroid. Previous studies have also observed that the outer retina, IS/OS junction, RPE, and vitreous are also affected by inflammation (Vasconcelos-Santos et al. 2010; Lee et al. 2009; Zhou et al. 2015). These findings indicate the existence of an immune mechanism against melanocytes accompanied by antigenic peptides accompany and managed by T-lymphocytes (Rao 1997).

Immunotherapy against superficial bladder cancer can be created by administration of BCG vaccine, which triggers a cytokine-mediated inflammatory response leading to the destruction of tumor cells (Uppal et al. 2010).

However, intravesical BCG therapy has been implicated in the development of anterior uveitis in several case reports. The mechanism behind this phenomenon is immune sensitization revealed by BCG with cross-reaction against the ocular antigen. Bilateral panuveitis (Jacob et al. 2006), chorioretinitis (Guex-Crosier et al. 2003), and/or optic neuritis (Hegde and Dean 2005) are rarely seen in patients, and mycobacterial choroiditis has been reported in only two previous case reports (Guex-Crosier et al. 2003).

There are two mechanisms that can be suggested for the origin of ocular inflammation: a local immune response, or a direct choroidal mycobacterial infection as demonstrated by vitreous cultures (Uppal et al. 2010). Burgoyne described the case of a 16-year-old Caucasian girl known not to have tuberculosis or any other systemic disease who developed acute panuveitis that progressed to bilateral serous retinal detachment after undergoing two sessions of purified protein derivative (PPD) skin testing separated by an interval of 8 years (Burgoyne et al. 1991).

A strong T-helper 1 response, which is known to be the most frequent type of immune response associated with uveitis, is induced by *M. tuberculosis*. Panuveitis is primarily caused by tuberculosis, VKH syndrome, sympathetic ophthalmia, Behcet’s disease, and sarcoidosis.

Uveitis, serous retinal detachment, and VKH may be potentially caused by antigenic mimicry. Investigation of the highest Th1 responses with the sequences of BCG proteins induced by the comparison of the amino acid sequences of retinal proteins and peptides identified several highly similar or even identical regions of 5–11 amino acids. Crossreactivity of the patient’s BCG-specific T cells with retinal autoantigen might have been caused by these epitopes. The previously reported uveitis was presumed to be caused by this phenomenon, referred to as antigenic mimicry (Garip et al. 2009).

BCG toxicity might be an expected result of immune stimulation and it proves that BCG is effective. Role of immune stimulation in the pathogenesis of VKH support this relationship.

EDI-OCT revealed our patients with acute VKH, increased choroidal thickness, RPE-Bruch membrane undulation, fluctuation of ILM, choroidal folds, retinal folds, vertical lining on the outer plexiform layer, increased choroidal hyperreflectivity, serous retinal detachment, Dalen-Fuchs nodules, and subretinal septa. To the best of our knowledge, this is the first report of DF nodules imaged by EDI-OCT in VKH. Previous study revealed focal collections of mononuclear inflammatory cells under elevated mounds of RPE led to the formation of Dalen-Fuchs nodules consisting of lymphocytes, pigment-laden macrophages, epithelioid cells, and proliferated RPE cells (Rao 2007).

The RPE undulation index was first described by Hosoda et al. (2014) and it was observed on the SD-OCT images that patients with VKH disease experienced significant distortions of the RPE layer. This distortion may be due to the choroidal congestion caused by the infiltration of inflammatory cells. Lin et al. (2014) first described the ILM fluctuations observed on the SD-OCT images of acute VKH patients. To the best of our knowledge, patient 2 is the second reported case of ILM fluctuation. Although the mechanism of ILM fluctuation is not fully known, inflammatory cells in vitreous have been hypothesized to cause local constriction of ILM, and retina edema to cause ILM fluctuation (Lin et al. 2014).

Clusters of T-lymphocytes activated around early choroidal melanocytes and macrophages accumulate abnormally and cause extensive choroidal thickening. VKH, in which choroid is the thickest tissue, is one of the main pathologies resulting from this phenomenon. In VKH, a significant increase in hyperreflective points is observed on an area of arterioles and venules, which are the medium choroidal vessels (Nazari et al. 2014). One of the most important findings of OCT in acute VKH disease is the large number of serous retinal detachment areas whose height of these may be much higher than those of serous retinal detachments in other tables. Although the fundus in the right eye of Patient 1 was normal at diagnosis, we found the choroid on EDI-OCT to be thick and observed increased choroidal hyperreflectivity, which indicates the changes concerning the disease. Abnormal increase in choroidal thickness causes fluctuations the RPE-Bruch membrane. The EDI-OCT images revealed that the IS/OS junction and RPE were mainly affected (Lee et al. 2009). These impacts included RPE distortions and disappearance of the IS/OS junction.

The length of the interval from the onset of symptoms to the initiation of the systemic steroid therapy is an important prognostic factor for patients with acute VKH, for whom proper and prompt treatment is crucial. Such treatment may prevent progression of the disease to the chronic recurrent stage and may possibly reduce the incidence and perhaps the severity of extraocular manifestations.

## Conclusion

Our findings indicate the importance of considering EDI-OCT findings, which allowed us to identify the histopathologic changes regarding the disease. To the best of our knowledge, patient 1 was the first documented case of VKH following BCG vaccination and patient 2 the first documented case of both VKH disease and tuberculosis. Based on our observations, we hypothesize that immunological mechanisms and dysregulation of the immune system may play a significant role in the association between VKH disease and BCG.

## References

[CR1] Burgoyne CF, Verstraeten TC, Friberg TR (1991). Tuberculin skin-test-induced uveitis in the absence of tuberculosis. Graefes Arch Clin Exp Ophthalmol.

[CR2] Garip A, Diedrichs-Mohring M, Thurau SR (2009). Uveitis in a patient treated with Bacille-Calmette–Guerin: possible antigenic mimicry of mycobacterial and retinal antigens. Ophthalmology.

[CR3] Guex-Crosier Y, Chamot L, Zografos L (2003). Chorioretinitis induced by intravesical Bacillus Calmette–Guerin (BCG) instillations for urinary bladder carcinoma. Klin Monbl Augenheilkd.

[CR4] Gupta V, Gupta A, Gupta P (2009). Spectral-domain cirrus optical coherence tomography of choroidal striations seen in the acute stage of Vogt–Koyanagi–Harada disease. Am J Ophthalmol.

[CR5] Hegde V, Dean F (2005). Bilateral panuveitis and optic neuritis following Bacillus Calmette–Guerin (BCG) vaccination. Acta Paediatr.

[CR6] Hosoda Y, Uji A, Hangai M (2014). Relationship between retinal lesions and inward choroidal bulging in Vogt–Koyanagi–Harada disease. Am J Ophthalmol.

[CR7] Ishihara K, Hangai M, Kita M (2009). Acute Vogt–Koyanagi–Harada disease in enhanced spectral-domain optical coherence tomography. Ophthalmology.

[CR8] Jacob M, Gambrelle J, Fleury J (2006). Panuveitis following intravesical bacille Calmette–Guerin therapy. J Fr Ophtalmol.

[CR9] Lee JE, Park SW, Lee JK (2009). Edema of the photoreceptor layer in Vogt–Koyanagi–Harada disease observed using high-resolution optical coherence tomography. Korean J Ophthalmol.

[CR10] Lin D, Chen W, Zhang G (2014). Comparison of the optical coherence tomographic characters between acute Vogt–Koyanagi–Harada disease and acute central serous chorioretinopathy. BMC Ophthalmol.

[CR11] Moorthy RS, Inomata H, Rao NA (1995). Vogt–Koyanagi–Harada syndrome. Surv Ophthalmol.

[CR12] Nazari H, Hariri A, Hu Z (2014). Choroidal atrophy and loss of choriocapillaris in convalescent stage of Vogt–Koyanagi–Harada disease: in vivo documentation. J Ophthalmic Inflamm Infect.

[CR13] Rao NA (1997). Mechanisms of inflammatory response in sympathetic ophthalmia and VKH syndrome. Eye.

[CR14] Rao NA (2007). Pathology of Vogt–Koyanagi–Harada disease. Int Ophthalmol.

[CR15] Read RW, Holland GN, Rao NA (2001). International Committee on Vogt–Koyanagi–Harada Disease Nomenclature. Revised diagnostic criteria for Vogt–Koyanagi–Harada disease: report of an international committee on nomenclature. Am J Ophthalmol.

[CR16] Read RW, Rechodouni A, Butani N (2001). Complications and prognostic factors in Vogt–Koyanagi–Harada disease. Am J Ophthalmol.

[CR17] Uppal GS, Shah AN, Tossounis CM (2010). Bilateral panuveitis following intravesical BCG immunotherapy for bladder carcinoma. Ocul Immunol Inflamm.

[CR18] Vasconcelos-Santos DV, Sohn EH, Sadda S (2010). Retinal pigment epithelial changes in chronic Vogt–Koyanagi–Harada disease: fundus autofluorescence and spectral domain-optical coherence tomography findings. Retina.

[CR19] Zhou M, Jiang C, Gu R (2015). Correlation between retinal changes and visual function in late-stage Vogt–Koyanagi–Harada disease: an optical coherence tomography study. J Ophthalmol.

